# rhCC16 Suppresses Cellular Senescence and Ameliorates COPD‐Like Symptoms by Activating the AMPK/Sirt1‐PGC‐1‐α‐TFAM Pathway to Promote Mitochondrial Function

**DOI:** 10.1111/jcmm.70566

**Published:** 2025-04-21

**Authors:** Ying‐jie Ren, Tian‐qi Sun, Yu Lu, Dan‐Li Liu, Rui Gao, Ting Li, Min Guo, Qing‐hua Liu, Hai‐long Wang, Min Pang

**Affiliations:** ^1^ NHC Key Laboratory of Pneumoconiosis; Shanxi Province Key Laboratory of Respiratory Disease; Department of Pulmonary and Critical Care Medicine The First Hospital Shanxi Medical University Taiyuan China; ^2^ School of Basic Medicine, Basic Medical Science Center Shanxi Medical University Jinzhong China; ^3^ School of Pharmacy, Academy of Medical Sciences Shanxi Medical University Jinzhong China; ^4^ Laboratory of Animal Center, Shanxi Key Laboratory of Experimental Animal Science and Animal Model of Human Disease, Shanxi Medical University Taiyuan China; ^5^ Translational Medicine Research Center Shanxi Medical University Taiyuan Shanxi China

**Keywords:** AMPK, cellular senescence, COPD, mitochondrial function, rhCC16

## Abstract

Chronic obstructive pulmonary disease (COPD) is a widespread lung disease marked by alveolar wall damage, leading to inflammation and fibrosis. Key risk factors include age, smoking, sex, and education, with smoking being the most crucial. These factors are globally consistent and linked with aging. Club cell secretory protein 16 (CC16), primarily secreted by non‐ciliated bronchial epithelial cells, is crucial for pulmonary health, offering anti‐inflammatory and antioxidant benefits. CC16 levels are notably reduced in COPD, suggesting its enhancement as a potential treatment. In this study, cellular senescence of BEAS‐2B cells was stimulated using cigarette smoke extract (CSE) and the function of recombinant human CC16 protein (rhCC16) in cellular senescence was assessed by detecting the levels of β‐galactosidase, p16, p21, ROS and the underlined mechanism was revealed by measuring mitochondrial biogenesis and metabolism. Additionally, COPD mice were prepared, and rhCC16's role on the cellular senescence of lung tissues was examined. Our findings showed that rhCC16 ameliorated cellular senescence in BEAS‐2B cells and lung tissues of COPD mice accompanied by lower levels of β‐galactosidase, p16, p21 and ROS. Mechanically, rhCC16 mitigated senescence via triggering PGC‐1α expression through the AMPK/SIRT1 pathway and fostering mitochondrial biogenesis and metabolism to reduce the levels of ROS. Furthermore, the results also indicated that rhCC16 exerted its effect via both integrin α4β1 and clathrin‐mediated endocytosis. Collectively, rhCC16 suppresses cellular senescence and ameliorates COPD‐like symptoms by activating the AMPK/Sirt1‐PGC‐1‐α‐TFAM pathway to foster mitochondrial function.

## 1 Introduction

Chronic obstructive pulmonary disease (COPD) is a common disease of the lung that causes irreversible damage to the alveolar wall and leads to inflammatory cell infiltration and fibrosis around the airways. The morbidity and fatality rates associated with COPD increase with age [1]. Recent studies conducted in China have revealed several independent risk factors for COPD, including age, history of smoking, sex, and education level, among others [2]. Similarly, in other countries, the above factors are also independent risk factors for COPD [3]. Among these risk factors, smoking is considered the most important. Moreover, with increasing age, cigarette susceptibility and clinical manifestations of the disease will progressively increase. Smoking, air pollution, occupational exposure, respiratory infections during childhood that are not properly treated (such as asthma and chronic bronchitis), and genetic factors such as human Hedgehog interacting protein gene polymorphisms can all lead to the development of COPD during the aging process [4, 5, 6]. COPD has many independent risk factors. Aging and cellular senescence alone do not cause COPD, but each independent factor is also somewhat connected to aging. Previous studies have reported a close relationship between aging and chronic lung disease, with the former now being recognised as an important driving mechanism of COPD [7]. The development of COPD also promotes cell senescence, leading to a vicious cycle of disease progression [8]. Therefore, exploring anti‐senescence strategies is important for developing treatment approaches for COPD.

Club cell secretory protein 16 (CC16), also referred to as CC10, is a secretory globulin or uterine globulin released mainly from club cells located on the mucosal lining of bronchioles and terminal bronchioles. The CC16 protein is encoded by the *SCGB1A1* gene. It is mainly secreted by non‐ciliated bronchial epithelial cells and is known for its anti‐inflammatory and antioxidative effects. CC16 contributes to pulmonary defence mechanisms by being involved in the secretion of surfactants and other protective substances. It plays a vital role in maintaining pulmonary health and homeostasis and is particularly important in the context of lung diseases such as COPD [9]. According to reports, the number of club cells and, consequently, the secretion of CC16 in the small airways of patients with COPD are decreased significantly compared to those in healthy subjects, in addition to the decreased CC16 content in the bronchoalveolar lavage fluid [10]. Therefore, CC16 augmentation is considered a potential strategy for COPD treatment [1]. However, only a few studies have been conducted on CC16, which have focused mainly on its anti‐inflammatory effect, with reports stating that CC16 effectively inhibits the accumulation of inflammatory factors during the inflammatory response [11], while reports on the anti‐senescence effect of CC16 are scarce.

Cellular senescence may be induced by a variety of stress factors, including DNA damage, epigenomic stress, mitochondrial dysfunction, and redox imbalance [12]. Mitochondria are the main organelles that produce intracellular reactive oxygen species (ROS). Mitochondrial dysfunction leads to ROS accumulation in cells, thereby aggravating oxidative stress, which is one of the initial factors leading to cell senescence [13]. Several recent studies have indicated that CC16 exhibits antioxidant effects [14, 15]. Therefore, it was hypothesised that the CC16 protein inhibits senescence by targeting mitochondria and thereby regulating cellular oxidative stress. The purpose of this study was to investigate the anti‐senescence effect of CC16 and uncover the underlying mechanism. Herein, experiments conducted using recombinant human CC16 protein (rhCC16) revealed that rhCC16 could promote mitochondrial biogenesis, oxidative metabolism, and an inflammatory phenotype, ultimately leading to suppressed cellular senescence in bronchial epithelial cells. The mechanism analysis revealed that rhCC16 induced PGC‐1α expression via the AMPK/SIRT1 signalling pathway through α4β1 integrin and clathrin‐mediated endocytosis and promoted mitochondrial functional biogenesis, thereby improving mitochondrial function and ultimately delaying cell senescence and COPD progression.

## 2 Materials and Methods

### 2.1 Chemicals and Reagents

The cigarettes were purchased from Yunnan Hong Ta Tobacco Co. Ltd. (Yunnan, China). The composition of each cigarette was as follows: 1.2 mg of nicotine, 13 mg of tar, and 13 mg of carbon monoxide. Dorsomorphin (an inhibitor of p‐AMPK) and Ex527 (an inhibitor of Sirt1) were purchased from Beyotime Biotechnology (Shanghai, China). Notably, while dorsomorphin is a potent adenosine 5'‐monophosphate (AMP)‐activated protein kinase (AMPK) inhibitor, it does not significantly inhibit other structurally related kinases, making it a useful tool for studying AMPK‐related pathways and processes. The clathrin inhibitor M‐β‐CD was purchased from Aladdin (Shanghai, China). BIO‐1211, an α4β1 inhibitor, was purchased from AbMole (Shanghai, China). The CCK8 Kit, SevenFast Total RNA Extraction Kit, All‐in‐One First Strand cDNA Synthesis Kit, and 2× SYBR Green qPCR Master Mix were purchased from Seven Biotech (Beijing, China). The primary and secondary antibodies used in the present study were as follows: anti‐GAPDH, anti‐PARP and anti‐Uteroglobin (CC16) antibodies purchased from Bioss (Beijing, China); anti‐CDKN2A/p16^INK4α^, anti‐CDKN1A/p21^CIP1^, anti‐Caspase3 and anti‐GLB1 antibodies purchased from ABclonal (Wuhan, China); anti‐NDUFB10, anti‐SDHA, and anti‐UQCRC2 antibodies purchased from Sangon Biotechnology (Shanghai, China); anti‐AMPK, anti‐p‐AMPK, anti‐Sirt1, anti‐PGC‐1α, and anti‐TFAM antibodies purchased from Beyotime Biotechnology (Shanghai, China); and HRP‐labelled goat anti‐rabbit/mouse IgG (H + L) purchased from Boster Biotechnology (Wuhan, China).

### 2.2 Cell Culture and Treatment

Human bronchial epithelial BEAS‐2B cells and a complete culture medium for BEAS‐2B cells were purchased from iCell Bioscience (Shanghai, China). The cells were cultured at 37°C in a 5% CO_2_ atmosphere. BEAS‐2B cells are often used in studies related to cellular senescence mechanisms because they are derived from the human bronchial epithelium and exhibit characteristics relevant to aging research. These cells provide a model for studying the effects of environmental factors, such as pollutants and cigarette smoke, which are known to accelerate aging processes in respiratory cells. Furthermore, their established genetic background and ability to be cultured easily make them suitable for conducting controlled experiments on cellular aging and senescence, particularly in the context of lung health and disease. The cellular senescence model was established through treatment with 5% CSE for 72 h, after which the cell culture supernatant and the cell pellet were collected and subjected to further analysis. First, the cells were pretreated with dorsomorphin (1 μM), Ex527 (10 μM), M‐β‐CD (5 μM) or BIO‐1211 (10 μM) for 6 h, followed by treatment with 250 ng/mL rhCC16 for 2 h and then with 5% CSE for another 72 h.

### 2.3 CSE Preparation

Cigarette smoke extract (CSE) was freshly prepared from a burning cigarette just prior to the treatments using the method described in a previous report [16].

### 2.4 Expression and Purification of rhCC16

The open reading frame (ORF) of the human CC16 gene was amplified from the complementary deoxyribonucleic acid (cDNA) of BEAS‐2B cells through reverse transcription‐polymerase chain reaction (RT‐PCR) conducted using a pair of specific primers. Next, the pGEX‐6P‐1/hCC16 plasmid was constructed, and the expression of the recombinant GST‐hCC16 protein in the host *E. coli* Transetta (DE3) cells was induced through treatment with 0.5 mM isopropyl β‐D‐1‐thiogalactopyranoside (IPTG) at 37°C for 6 h. The levels of GST‐hCC16 in different cellular components were measured using sodium dodecyl sulfate‐polyacrylamide gel electrophoresis (SDS‐PAGE) followed by Coomassie blue R‐250 staining and Western blot.

The obtained GST‐hCC16 was purified using a self‐packaged glutathione S‐transferase (GST)‐affinity column packaged with 2 mL of BeyoGold GST‐tag Purification Resin (Beyotime, China). To remove the GST tag from the protein, the purified protein was applied to a column loaded with PreScission protease (Beyotime, China) at 4°C and left undisturbed for the entire night, after which it was applied to a Superdex‐75 (Pharmacia, USA) column. The purified rhCC16 was then collected and concentrated using an Amicon Ultra‐3000 MWCO (Millipore, USA). The concentrated protein was subjected to SDS‐PAGE followed by Coomassie blue R‐250 staining. The endotoxin contaminants in the obtained rhCC16 were removed and collected using the ToxinEraser Endotoxin Removal Kit and measured using the Chromogenic End‐point Endotoxin Assay Kit (Chinese Horseshoe Crab Reagent Manufactory, China). The rhCC16 fraction with an endotoxin content less than 0.1 EU/mL was filtered through a 0.2‐μm pore membrane, and the bioactivity of the resulting filtrate was assessed via acid–base titration analysis according to previously published protocols [17].

### 2.5 Cell Viability Assay

BEAS‐2B cells were seeded in the wells of a 96‐well plate at a density of 2 × 10^3^/well and then treated with rhCC16 or CSE as indicated. Later, the spent medium was removed, after which the cells were incubated in 100 μL of medium containing 10 μL of CCK‐8 solution at 37°C for 1 h. Finally, the absorbance of each well was measured at 450 nm using a microplate reader (Eppendorf, Germany).

### 2.6 Measurement of Intracellular ROS

The intracellular reactive oxygen species (ROS) concentration was detected with a 2,7‐dichlorodihydrofluorescein diacetate (DCFH‐DA) probe (ServiceBio, Wuhan, China) according to the manufacturer's instructions using flow cytometry (FACSCelesta flow cytometry instrument, Becton Dickinson). Additionally, the fluorescence intensity was also measured under a fluorescence microscope (Leica‐DM5000, Germany) and quantified using a fluorescence plate reader (BMG Labtech, FLUO star OPTIMA) at excitation and emission wavelengths of 488 nm and 525 nm, respectively.

### 2.7 Measurement of mtROS Levels

Mitochondrial ROS (mtROS) levels were tested with the MitoSOX Red Mitochondrial Superoxide Indicator dye (Yeasen Biotechnology, Shanghai, China) according to the manufacturer's instructions using flow cytometry (FACSCelesta flow cytometry instrument, Becton Dickinson). Additionally, the MitoSOX fluorescence intensity was also detected under a fluorescence microscope and quantified using a fluorescence plate reader at excitation and emission wavelengths of 510 nm and 580 nm, respectively.

### 2.8 ATP Detection

The adenosine triphosphate (ATP) levels were determined using an ATP Assay Kit purchased from Beyotime (Shanghai, China) according to the manufacturer's protocols. BEAS‐2B cells were treated as described above, and total protein was extracted from these cells. The protein concentrations were measured using a bicinchoninic acid (BCA) assay kit (Abbkine, Wuhan, China). The ATP levels were then determined using a luminometer (Spectra Max ID5, Molecular Devices, USA) and are expressed in nmol/mg of protein.

### 2.9 NAD ^+^/NADH and CC16 Assays

The NAD^+^ and nicotinamide adenine dinucleotide (NADH) levels were determined using an NAD^+^/NADH assay kit from Beyotime (Shanghai, China) according to the manufacturer's instructions. The levels of CC16 protein in the lung tissues of the mice were determined using an ELISA kit from Bioswamp (Wuhan, China), with a sensitivity of ≤ 12.5 pg/mL and a detection range of 62.5 pg/mL to 5000 pg/mL.

### 2.10 Senescence‐Associated β‐Galactosidase Staining

Senescence‐associated β‐galactosidase (SA‐β‐gal) staining was performed according to the manufacturer's instructions (ServiceBio, Wuhan, China). The blue‐stained senescent cells were detected under a microscope (OLYMPUS, Japan). The percentage of stain‐positive cells was determined using ImageJ software.

### 2.11 Hoechst33258 Staining

BEAS‐2B cells were seeded at a density of 1 × 10^4^ cells per well in a 12‐well plate with a cell slide, incubated overnight, and treated with CSE (0%, 2.5%, 5%) for 72 h. The cells were washed with phosphate buffered saline (PBS) and incubated with 5% bovine serum albumin (BSA) at room temperature for 30 min. The cells were then washed twice with PBS, stained with Hoechst33258 for 10 min and imaged under a fluorescence microscope (Olympus Corporation, Tokyo, Japan). Five random fields in each well were imaged and analysed to determine the mean fluorescence intensity (MFI) with ImageJ software.

### 2.12 Real‐Time PCR

Total ribonucleic acid (RNA) was extracted from the cells using a Total RNA Extraction Kit. The obtained RNA was subjected to cDNA synthesis using the All‐in‐One First Strand cDNA Synthesis Kit, followed by RT–PCR using 2× SYBR Green qPCR Master Mix. The primers used for PCR are listed in Table 1. β‐Actin was used as the housekeeping gene for q‐PCR normalisation. The gene expression levels were quantified using the comparative Ct method (2^−ΔΔCt^), followed by data analysis.

**TABLE 1:** Primers sets used for real‐time PCR.

Gene	Sense primer (5′–3′)	Antisense primer (5′–3′)
Human *p16*	GGGTTTTCGTGGTTCACATCC	CTAGACGCTGG CTCCTCAGTA
Human *p21*	CGATGGAACTTCGACTTTGTCA	GCACAAGGGTACAAGACAG TG
Human *SOD1*	GGTGGGCCAAAGGATGAAGAG	CCACAAGCCAAACGACTTCC
Human *SOD2*	GCTCCGGTTTTGGGGTATCTG	GCGTTGATGTGAGGTTCCAG
Human *CAT*	TGGAGCTGGTAACCCAGTAGG	CCTTTGCCTTGGAGTATTTGGTA
Human *GPX‐4*	GAGGCAAGACCGAAGTAAACTAC	CCGAACTGGTTACACGGGAA
Human *IL‐6*	TCCTCTCCACAAACATGTAACAA	TCACCAGGCAAGTCTCCTCA
Human *IL‐8*	TTTTGCCAAGGAGTGCTAAAGA	AACCCTCTGCACCCAGTTTTC
Human *IL‐1α*	TGGTAGTAGCAACCAACGGGA	ACTTTGATTGAGGGCGTCATTC
Human *IL‐1β*	ATGATGGCTTATTACAGTGGCAA	GTCGGAGATTCGTAGCTGGA
Human *CXCL‐1*	ACTGCACCCAAACCGAAGTC	TGGGGACACCTTTTAGCATCTT
Human *CCL‐2*	CAGC CAGATGCAATCAATGCC	TGGAATCCTGAACCCACTTCT
Human *MMP‐1*	AAAATTACACGCCAGA TTTGCC	GGTGTGACATTAACTCCAGAGTTG
Human *MMP‐3*	CGGTTCCGCCTGTCTCAAG	CGCCAAAAGTGCCTGTCTT
Human *β‐Actin*	CATGTACGTTGCTATCCAGGC	CTCCTTAATGTCACG CACGAT

### 2.13 Western blot Analysis

Briefly, BEAS‐2B cells were lysed in SDS lysis buffer, and the concentration of the released protein was measured using the BCA method. The protein was then subjected to electrophoresis using a 12% gel and a mass loading of 30 μg per well. Next, the proteins were transferred to a polyvinylidene fluoride (PVDF) membrane and then blocked with 5% BSA for 1 h at room temperature. The membrane containing the proteins was then incubated overnight with the relevant primary antibodies at 4°C, after which the protein bands were visualised using an enhanced chemiluminescence (ECL) Western blot analysis system (Alpha Imager, MINI, USA). The intensity of each protein band was determined using ImageJ software. The relative levels of the indicated proteins were determined using optical densitometry and normalised to the level of GAPDH as a reference.

### 2.14 Animal Model and Treatment

Six‐week‐old male C57BL/6 mice were procured from the Experimental Animal Center of Shanxi Medical University (Shanxi, China). All mice were housed under a 12‐h light/12‐h dark photoperiod and specific pathogen‐free conditions with access to unlimited food and water. All animal experiments were performed in accordance with the guidelines of the Animal Ethics Committee of Shanxi Medical University (SYDL2021016) and the National Institutes of Health Guide for the Care and Use of Laboratory Animals (NIH publication no. 85–23, revised 1996) [18].

The mice were divided into three groups, each comprising 10 mice: the control, COPD, and rhCC16 treatment groups. The mice in the COPD and treatment groups were placed inside a semi‐closed smoking box (capacity: 27 L) where they were exposed to smoke from 10 commercial filtered cigarettes (Hongtashan, Yunnan, China) for 2 h a day, and this inhalation experiment was conducted 5 days a week for 24 weeks. The mice in the control group were allowed to breathe normal air. In the last month prior to the end of the feeding experiment, all mice in the treatment group were intranasally administered rhCC16 (2.5 μg/g body weight) 2 h before smoking every day for 30 days, while the mice in the COPD and control groups received daily intranasal inhalation of an equal volume of sterile PBS.

### 2.15 Pulmonary Function Test

The pulmonary function of the mice was evaluated using a pulmonary function testing system (PFT‐MR, Shanghai, China) according to the manufacturer's protocol. The system collected data regarding the ratio of forced expiratory volume in 100 ms to forced vital capacity (FEV_100_/FVC), 10% forced expiratory flow (FEF10) (mL/s), dynamic lung compliance Cdyn (mL/cmH20), and resistance (R) (cmH20*s/mL), following which the obtained data regarding these parameters were analysed to determine the effects of cigarette smoke (CS) exposure and rhCC16 treatment.

### 2.16 Immunohistochemistry (IHC) Analysis

IHC staining was performed following standard protocols [19]. The primary antibodies used in the Western blot analysis were also used in the immunohistochemistry analysis, while conjugated horseradish peroxidase from Beijing Zhongshan Jinqiao Biotechnology Co. Ltd. (Beijing, China) was used as the secondary antibody in this analysis. When each sample was examined under a microscope (Olympus BX61, Japan), three fields of view were randomly selected at 400x magnification. The IHC analysis plugin of ImageJ analysis software was used to determine the percentage of the total image area that was occupied by positively stained tissues (brown areas) in representative immunohistochemical images. This is expressed as a percentage area fraction. It can reflect the levels of various indicators expressed in lung tissue.

### 2.17 Statistical Analysis

The resulting data were analysed statistically using GraphPad Prism software (GraphPad Software, CA, USA). The data were presented as the mean ± SEM. One‐way ANOVA was performed to compare multiple groups. A *t*‐test was conducted to compare two groups. *p* < 0.05 was considered to indicate statistical significance. The results of each in vitro experiment were derived from at least three repeated experiments; the analysis results of in vivo experiments were obtained from three or more different individuals.

## 3 Results

### 3.1 Preparation of rhCC16

**FIGURE 1:**
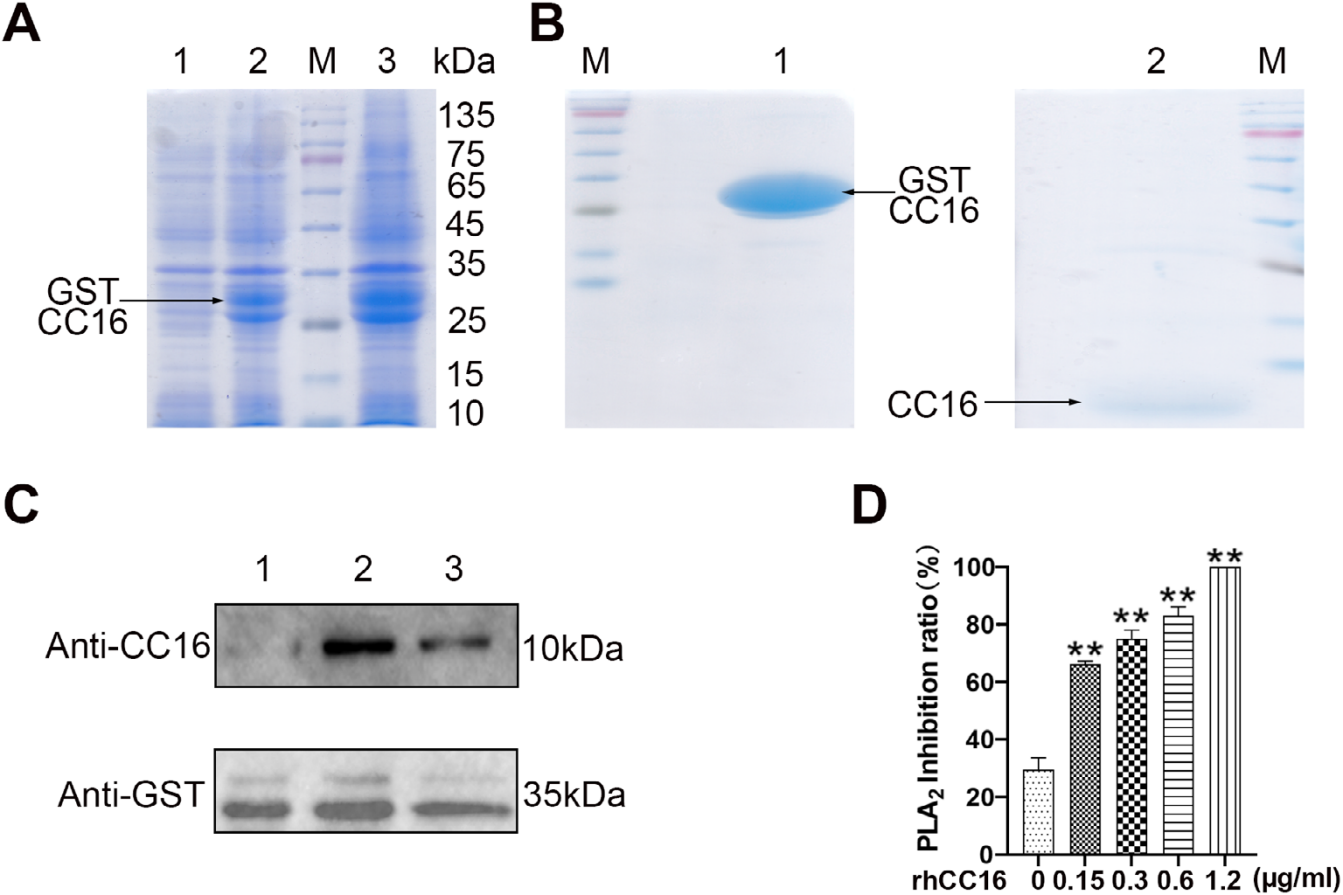
Preparation of rhCC16. (A) Induction of GST‐hCC16 in the host *Escherichia coli* strain using IPTG. Lane 1: Uninduced protein; Lane 2: Induced total protein; Lane 3: The protein in the supernatant. (B) Lane 1: GST‐rhCC16 bound to the solid‐phase carrier after the removal of contaminants. Lane 2: Purified rhCC16 obtained after enzymatic digestion of GST. (C) Verification of the obtained GST‐rhCC16 using Western blot. (D) Evaluation of the bioactivity of rhCC16 using acid–base titration. The data were presented as the mean ± SEM. *p < 0.05 and **p < 0.01, compared to the control group.

The pGEX‐6P‐1/hCC16 plasmid was constructed and transformed into *E. coli* Transetta (DE3). GST‐hCC16 was induced using 0.5 mM IPTG (Figure 1A) and verified through Western blot using CC16 or GST primary antibodies (Figure 1C). The GST tag in GST‐hCC16 was then removed through PreScission Protease digestion on a glutathione S‐transferase (GST)‐affinity column, and the purified rhCC16 thus obtained was subjected to verification through Coomassie blue R‐250 staining (Figure 1B). Finally, an acid–base titration experiment was conducted and revealed that rhCC16 inhibited the activity of phospholipase A_2_ in a dose‐dependent manner (Figure 1D). This finding indicated that rhCC16 exhibited biological activity.

### 3.2 rhCC16 Ameliorated Both Cellular Senescence in Lung Tissues and COPD‐Like Pathological Changes in COPD Mice

**FIGURE 2:**
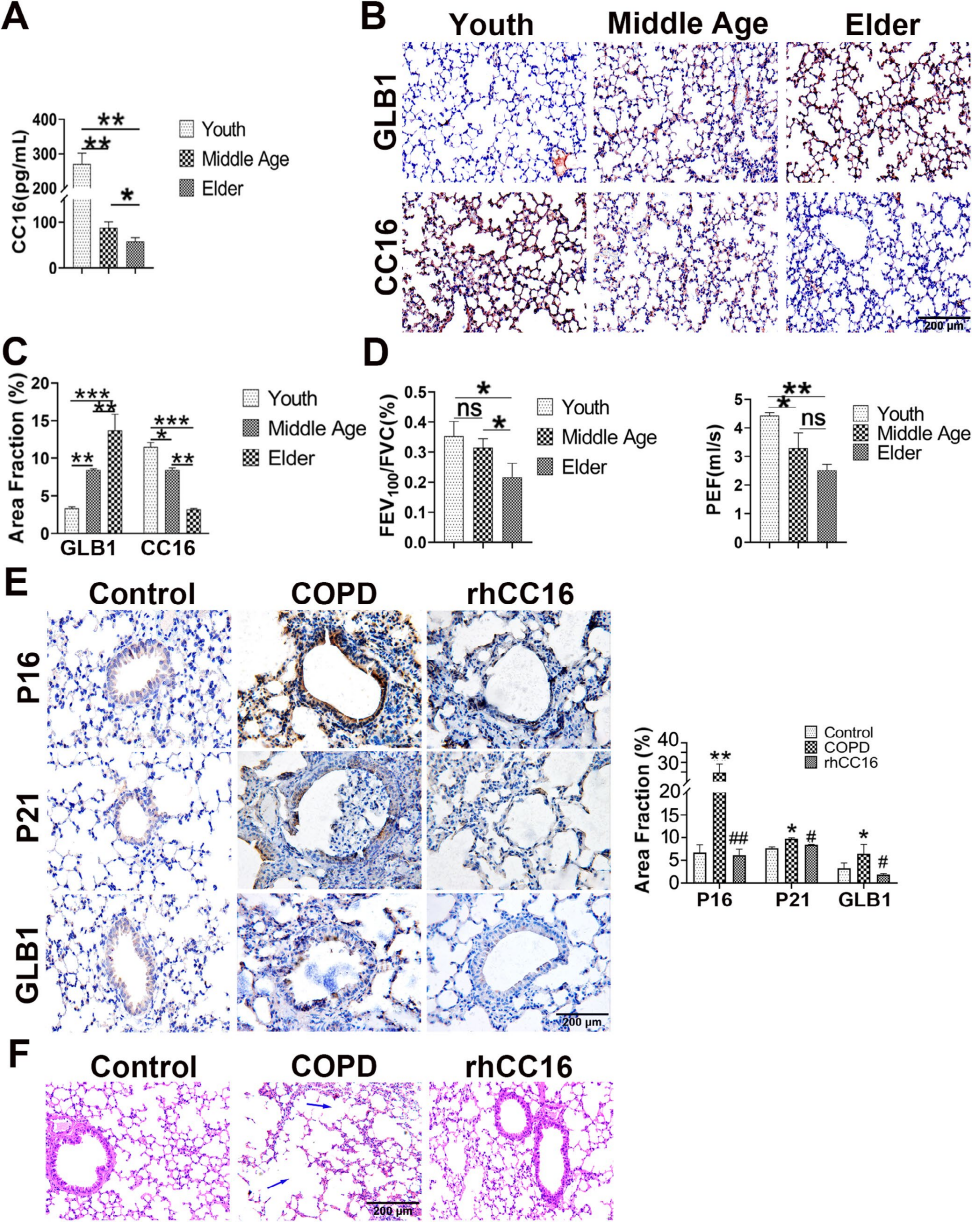
Treatment with rhCC16 ameliorated cellular senescence in mouse lung tissue cells and COPD‐like pathological changes in mice with COPD. (A) The levels of CC16 protein in the lung tissues of mice at different ages were measured using ELISA. (B) Representative immunohistochemical images of CC16 and GLB1 in the lung tissues of mice; scale bar: 100 μm. (C) The statistical graph of the data obtained from the immunohistochemistry analysis presented in (B) were expressed as positive cell area fractions. (D) Results of lung function tests conducted with mice at different ages. (E) Representative images of P16, P21, and GLB1 obtained by immunohistochemical analysis in the control, COPD, and rhCC16 groups; scale bar: 200 μm. (F) Representative images of H&E‐stained mouse lung tissue samples from the control, COPD, and rhCC16 groups. The mice in the COPD group and rhCC16 group were exposed to smoke for 24 weeks, 5 days a week, for 2 h each day. In the 21st week, the mice in the rhCC16 group were treated with intranasal administration 2 h before smoke exposure, and the treatment lasted for 1 month. After 24 weeks, fresh lung tissues were collected, embedded, and paraffin sections were prepared for HE staining. The data were presented as the mean ± SEM. *p < 0.05 and **p < 0.01, ***p < 0.001, compared to the control group; ^#^ *p* < 0.05, ^##^ *p* < 0.01, compared to the COPD group.

First, the CC16 protein content in the lungs of C57BL/6 mice gradually decreased with increasing age, while the levels of beta galactosidase 1 (GLB1), which is a marker of cellular senescence, gradually increased (Figure 2A–C). The decreases in the lung function expiratory indices FEV_100_/FVC and PEF also suggested that older mice were more sensitive to COPD (Figure 2D). Next, a COPD mouse model was established, and the results revealed that rhCC16 augmentation reduced the expression of P16, P21, and GLB1 in the lung tissue of these mice with COPD (Figure 2E). Moreover, COPD‐like pathological changes, such as alveolar wall rupture, alveolar fusion, and inflammatory cell infiltration, were ameliorated in the rhCC16 treatment group (Figure 2F). In addition, rhCC16 administration promoted pulmonary function in mice with COPD (Table 2). Collectively, these findings demonstrated that exogenous supplementation with rhCC16 could alleviate cellular senescence in lung tissues and promote lung function in mice with COPD.

**TABLE 2:** Detection of lung function in different mice groups ($\bar{x}$±s, *n* = 10).

	FEV_100_/FVC	FEF10 (mL/s)	Cdyn (mL/cmH20)	R (cmH20 s/mL)*
Control	0.85 ± 0.06	0.1 ± 0.002	0.007 ± 0.0005	10.97 ± 0.32
COPD	0.54 ± 0.05*	0.04 ± 0.003*	0.004 ± 0.0005*	29.74 ± 7.10*
rhCC16	0.75 ± 0.08^#^	0.1 ± 0.002^#^	0.007 ± 0.0005^#^	13.51 ± 0.89^#^

* *p <* 0.05 vs. Control.

^*#*^ *p <* 0.05 vs. COPD.

### 3.3 rhCC16 Inhibited the Senescence of BEAS‐2B Cells Induced by CSE

**FIGURE 3:**
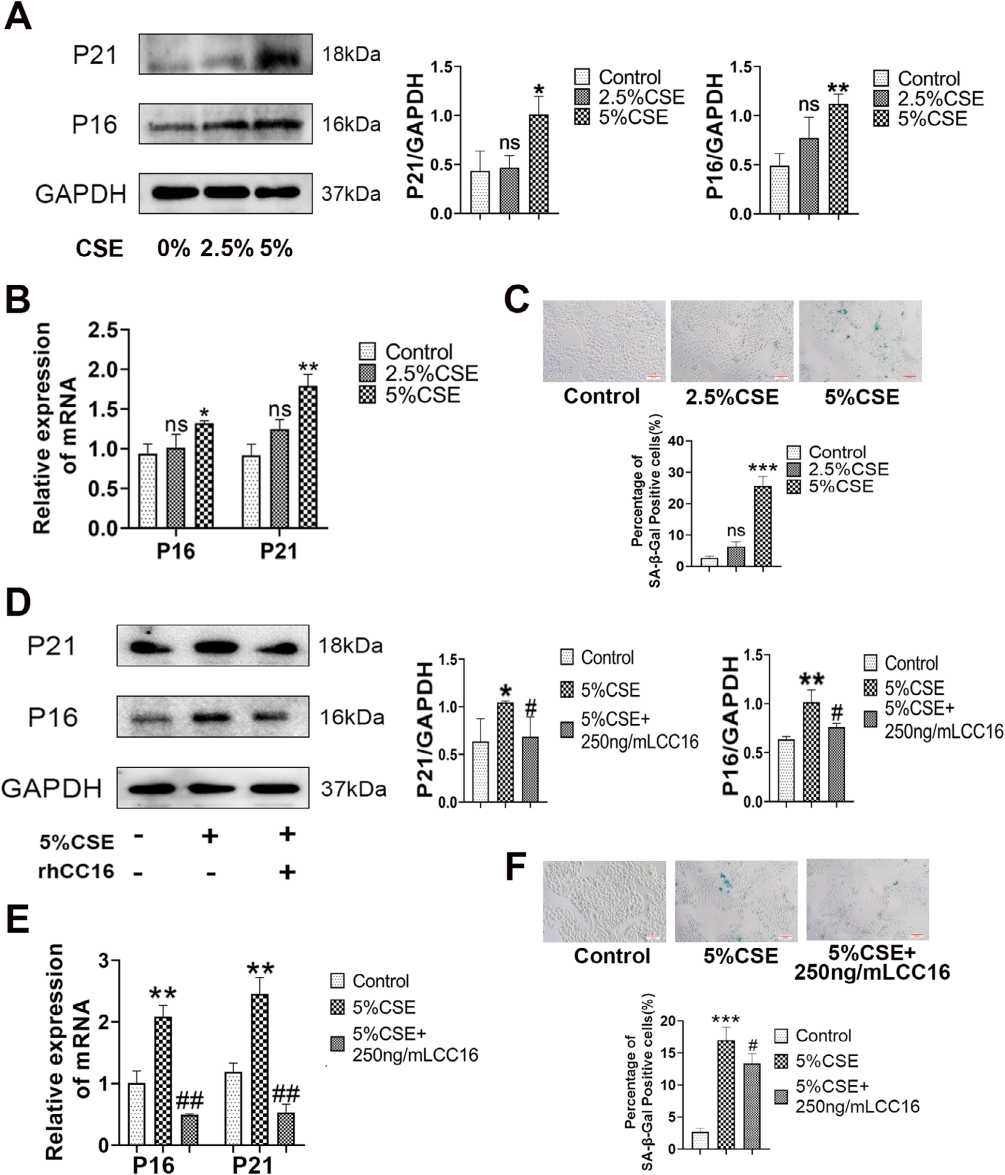
rhCC16 inhibited the senescence induced by CSE. (A) The protein levels of P16 and P21 in CSE‐stimulated BEAS‐2B cells were determined using Western blot. (B) The mRNA expression of *P16* and *P21* in CSE‐stimulated BEAS‐2B cells was determined through RT–qPCR. (C) Representative images of the senescence‐associated β‐galactosidase staining experiment conducted with CSE‐stimulated BEAS‐2B cells; scale bar: 20 μm. (D) The protein levels of P16 and P21 in CSE‐stimulated or rhCC16‐treated BEAS‐2B cells were determined using Western blot. (E) The mRNA expression of *P16* and *P21* in CSE‐stimulated or rhCC16‐treated BEAS‐2B cells was determined through RT–qPCR. (F) Representative images of senescence‐associated β‐galactosidase staining in CSE‐stimulated or rhCC16‐treated BEAS‐2B cells; scale bar: 20 μm. The data were presented as the mean ± SEM. *p < 0.05, **p < 0.01, and ***p < 0.001, compared to the control group; ^#^ *p* < 0.05, ^##^ *p* < 0.01, compared to the 5% CSE group.

To explore the mechanism underlying the anti‐aging effect of rhCC16, in vitro experiments were conducted using the human bronchial epithelial cell line BEAS‐2B. First, BEAS‐2B cells were treated with different concentrations of CSE for different durations. Next, the cells were subjected to the CCK8 assay, which revealed that treatment with 2.5% CSE and 5% CSE for 3 days ensured that the percentage of inhibited cells remained less than 50% (Figure S1A,B). The results of Western blot and qPCR analyses revealed that 2.5% CSE could not induce cellular senescence, while 5% CSE could enhance the levels of P16 and P21 (Figure 3A,B). This finding was also corroborated by the aging‐related β‐galactosidase (SA‐β‐Gal) staining test (Figure 3C). Additionally, the apoptosis of BEAS‐2B cells treated with 2.5% or 5% CSE for 3 days was measured using Western blot and Hoechst 33258 staining, and the results showed that BEAS‐2B cells did not undergo apoptosis (Figure S2A,B). The optimum concentration of rhCC16 was also determined using the same method and was determined to be 250 ng/mL (Figure S1C,D). Treatment of BEAS‐2B cells stimulated with 5% CSE and 250 ng/mL rhCC16 reduced the levels of P16 and P21 induced by CSE (Figure 3D,E). The SA‐β‐Gal staining results also confirmed the anti‐senescence effect of rhCC16 (Figure 3F).

### 3.4 rhCC16 Restrained Cellular Senescence by Reducing ROS Production Induced by CSE

**FIGURE 4:**
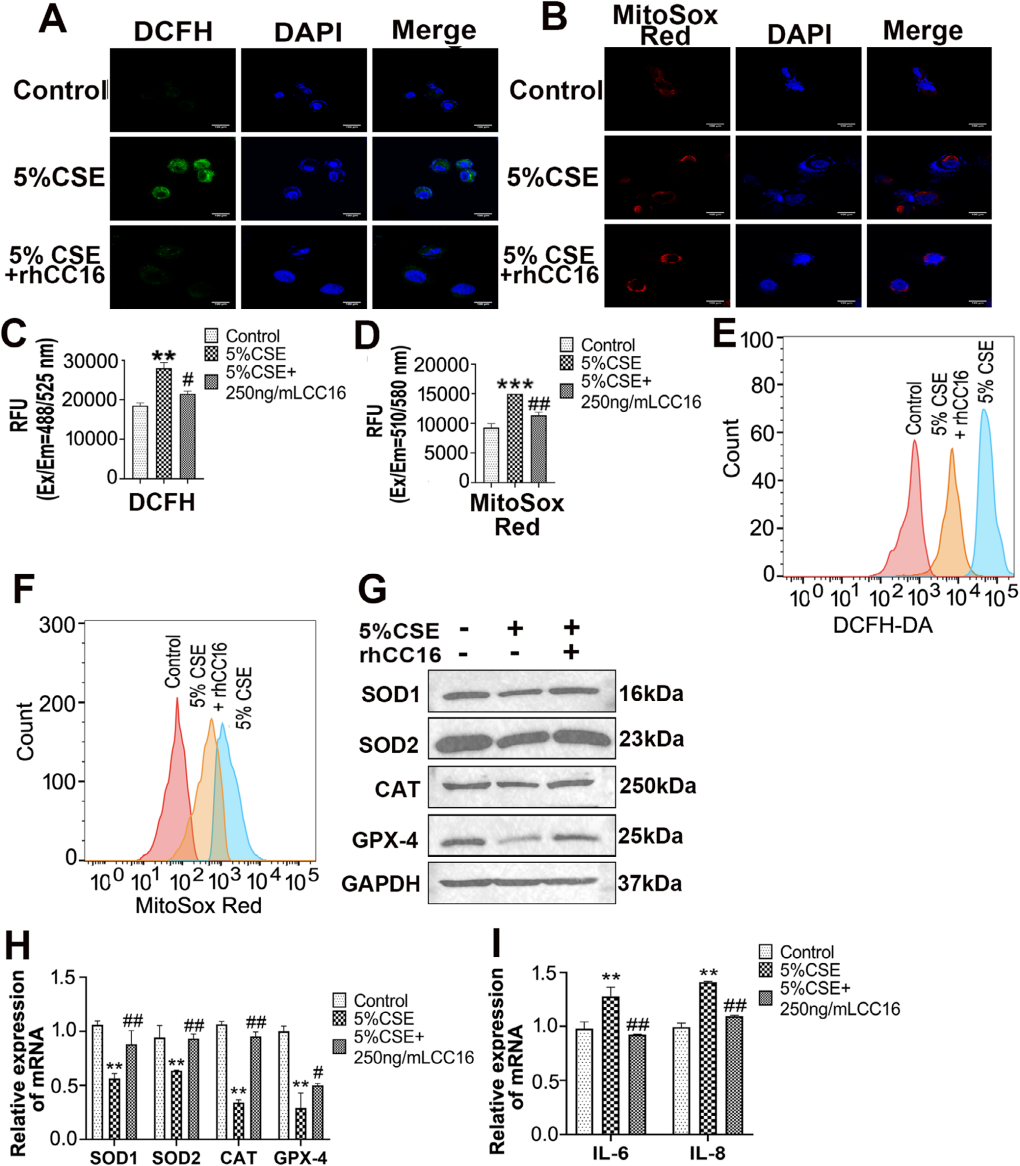
rhCC16 restrained cellular senescence by reducing ROS production induced by CSE. (A) Representative images of the detection of ROS levels using a DCFH probe (green) and nuclei stained with DAPI (blue); scale bar: 100 μm. (B) Representative images of the detection of mitochondrial ROS levels using the MitoSOX Red probe (red) and nuclei stained with DAPI (blue); scale bar: 100 μm. (C) The fluorescence intensity was calculated using a multifunctional microplate reader at an excitation wavelength of 488 nm and an emission wavelength of 525 nm. (D) The fluorescence intensity was calculated using a multifunctional microplate reader at an excitation wavelength of 510 nm and an emission wavelength of 580 nm. (E) Flow cytometry was used to observe the changes in the intracellular total ROS levels. (F) Flow cytometry was used to observe the changes in the mitochondrial ROS levels. (G) Western blot was used to determine the protein expression levels of SOD1, SOD2, CAT, and GPX‐4. (H) The mRNA expression levels of *SOD1*, *SOD2*, *CAT*, and *GPX‐4* were determined through RT–qPCR. (I) The mRNA expression levels of *IL‐6* and *IL‐8* were determined through RT–qPCR. The data were presented as the mean ± SEM. *p < 0.05 and **p < 0.01, ***p < 0.001 compared to the control group; ^#^ *p* < 0.05, ^##^ *p* < 0.01, compared to the 5% CSE group.

It is well recognised that reactive oxygen species (ROS) produced in mitochondria play a vital role in regulating cellular senescence. Therefore, the total ROS in the cells and the ROS production in the mitochondria were evaluated in the present study using DCFH and MitoSox Red probes, respectively, and the results were presented in Figure 4. Both total intracellular ROS and mitochondrial ROS increased significantly after treatment with 5% CSE, and after treatment with rhCC16, the levels of both decreased significantly (Figure 4A–F). These findings indicated that rhCC16 might be involved in the regulation of senescence in BEAS‐2B cells through the effective scavenging of ROS. To confirm this inference, Western blot and qPCR were performed to detect the expression of the antioxidant enzymes involved in ROS scavenging within mitochondria. After rhCC16 treatment, the gene and protein levels of *SOD1*, *SOD2*, *CAT*, and *GPX‐4*, which were downregulated by CSE stimulation in the cells, were restored to normal ranges (Figure 4G,H). Importantly, the addition of rhCC16 significantly reduced the extent of the senescence‐related secretory phenotype (SASP) induced by ROS accumulation (Figure 4I, Figure S3A). Collectively, these results demonstrated that rhCC16 inhibited cellular senescence by inhibiting ROS production induced by CSE.

### 3.5 rhCC16 Cleared ROS Accumulation in Mitochondria by Regulating Mitochondrial Function

**FIGURE 5:**
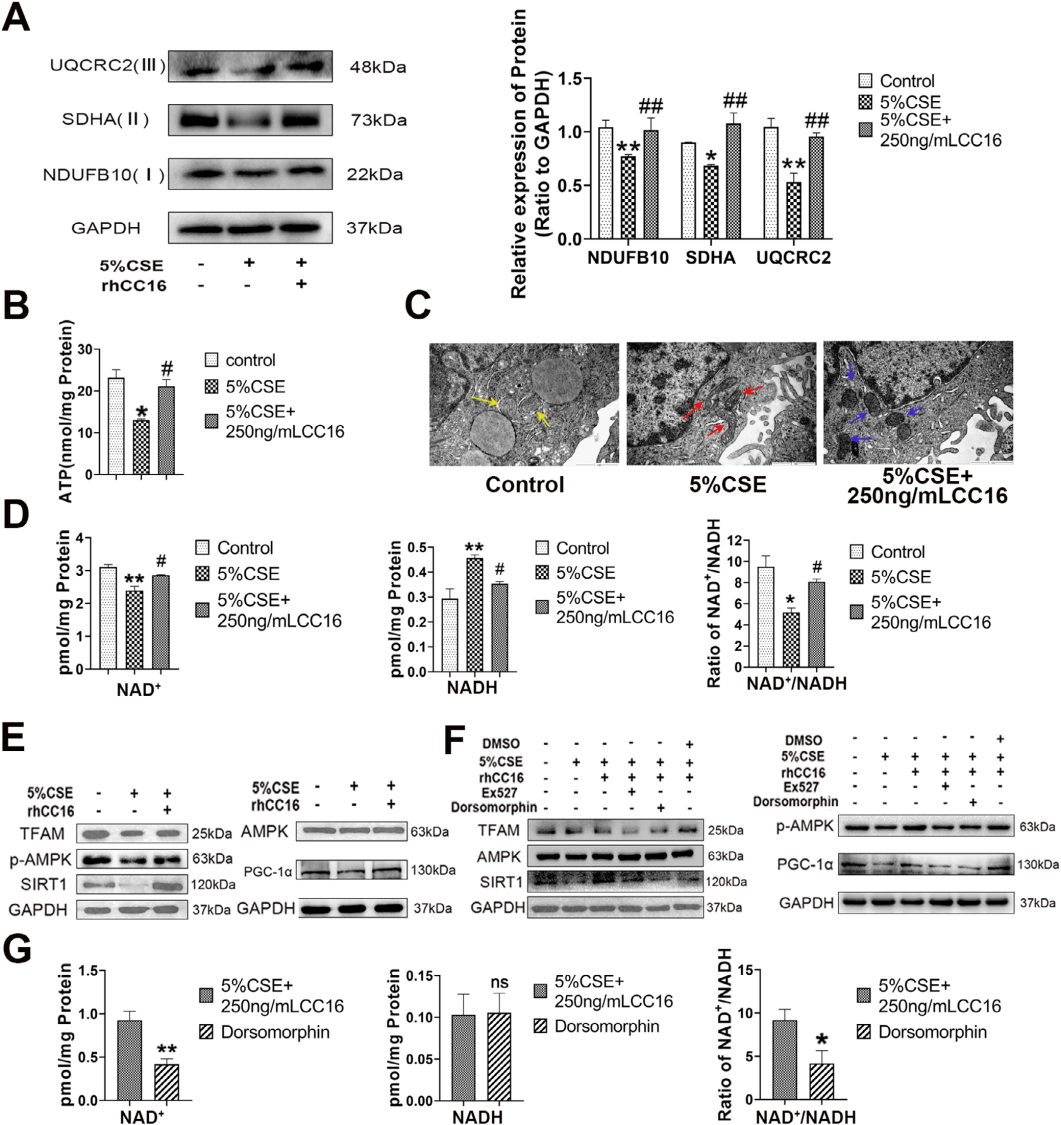
rhCC16 inhibited ROS accumulation by regulating mitochondrial function. (A) The levels of the oxidative phosphorylation complex I, complex II, and complex III proteins were determined using Western blot. (B) The intracellular ATP levels were determined using an ATP assay kit. (C) The ultrastructure of intracellular mitochondria in CSE‐stimulated or rhCC16‐treated BEAS‐2B cells, as observed under a transmission electron microscope; yellow arrows represent mitochondria with normal morphology; red arrows represent mitochondria with abnormal morphology and functional damage; blue arrows represent mitochondria with restored morphology and function. Scale bar: 1 μm. (D) The levels of NAD^+^ and NADH in CSE‐stimulated or rhCC16‐treated BEAS‐2B cells, based on which the NAD^+^/NADH ratio was calculated. (E) The levels of p‐AMPK, AMPK, SIRT1, PGC‐1α, and TFAM proteins in CSE‐stimulated or rhCC16‐treated BEAS‐2B cells were determined using Western blot. (F) The levels of p‐AMPK, AMPK, SIRT1, PGC‐1α, and TFAM proteins in CSE‐stimulated or rhCC16‐treated BEAS‐2B cells treated with Ex527 (10 μM) or dorsomorphin (1 μM) were determined using Western blot. (G) The intracellular concentrations of NAD^+^ and NADH in CSE‐stimulated or rhCC16‐treated BEAS‐2B cells treated with dorsomorphin (1 μM), based on which the NAD^+^/NADH ratio was calculated. The data were presented as the mean ± SEM. *p < 0.05 and **p < 0.01, compared to the control group; ^#^ *p* < 0.05, ^##^ *p* < 0.01 compared to the 5% CSE group.

A failure in the normal clearance of dysfunctional mitochondria results in abnormal accumulation of ROS, thereby promoting senescence [20]. Oxidative stress reportedly reduces the levels of oxidative phosphorylation (OXPHOS) [21]. Therefore, whether rhCC16 can reduce ROS production by improving mitochondrial function deterioration in CSE‐stimulated BEAS‐2B cells was investigated in the present study. To achieve this objective, the levels of the markers of complexes formed during mitochondrial oxidative phosphorylation (Complex I: NDUFB10, Complex II: SDHA, and Complex III: UQCRC2) were determined, and the results revealed significant downregulation of all three complex markers in CSE‐stimulated cells. Treatment with rhCC16 significantly increased the levels of all three markers (Figure 5A). In addition, rhCC16 administration restored the production of ATP, which decreased upon CSE stimulation (Figure 5B). Finally, to intuitively understand the conditions of the mitochondria, transmission electron microscopy was employed, which revealed that the intracellular mitochondria in CSE‐stimulated BEAS‐2B cells were thinner and longer, with irregular shapes, a disordered arrangement of internal cristae, and vacuoles with low electron density. This observation suggested that the mitochondrial content had decomposed, which was consistent with the previously reported structural state of mitochondria in senescent cells [22]. The rhCC16 intervention partially restored the structure of the mitochondria and the internal electronic density (Figure 5C). In summary, rhCC16 could suppress cellular senescence by improving the function of mitochondria and clearing ROS.

### 3.6 rhCC16 Improved Mitochondrial Function via the AMPK/Sirt1/PGC‐1α/TFAM Pathway Both in vitro and in vivo

Nicotinamide adenine dinucleotide (NAD^+^) plays a central role in energy metabolism and influences, either directly or indirectly, several key processes occurring within a cell, including cellular senescence [23]. NAD^+^ levels decrease when mitochondrial function is disrupted [24]. Therefore, in the present study, the concentration of intracellular NAD^+^ was determined, and rhCC16 treatment restored the concentration of intracellular NAD^+^ and the NAD^+^/NADH ratio, both of which were reduced upon CSE stimulation in BEAS‐2B cells (Figure 5D).

The AMPK/Sirt1/PGC‐1α/mitochondrial transcription factor A (TFAM) signalling pathway reportedly participates in the modulation of mitochondrial biogenesis, mitochondrial energy metabolism, and oxidative stress [25]. The NAD^+^ deacetylase Sirt1 is activated during cellular senescence and mitochondrial stress [26]. In the present study, we explored whether rhCC16 affects mitochondrial function through the AMPK/Sirt1/PGC‐1α/mitochondrial transcription factor A (TFAM) signalling pathway. Western blot analysis revealed that rhCC16 treatment increased the levels of phosphorylated AMPK (p‐AMPK), p‐Sirt1, PGC‐1α, and TFAM, which were reduced upon CSE stimulation in cells (Figure 5E, Figure S3B). Further experiments using dorsomorphin (an inhibitor of p‐AMPK) or Ex527 (an inhibitor of Sirt1) revealed that both AMPK and Sirt1 inhibition reversed the increased levels of PGC‐1α and TFAM observed after rhCC16 treatment (Figure 5F, Figure S3C). Moreover, when AMPK was suppressed with dorsomorphin, the concentration of NAD^+^ and the NAD^+^/NADH ratio decreased in rhCC16‐treated BEAS‐2B cells (Figure 5G).

**FIGURE 6:**
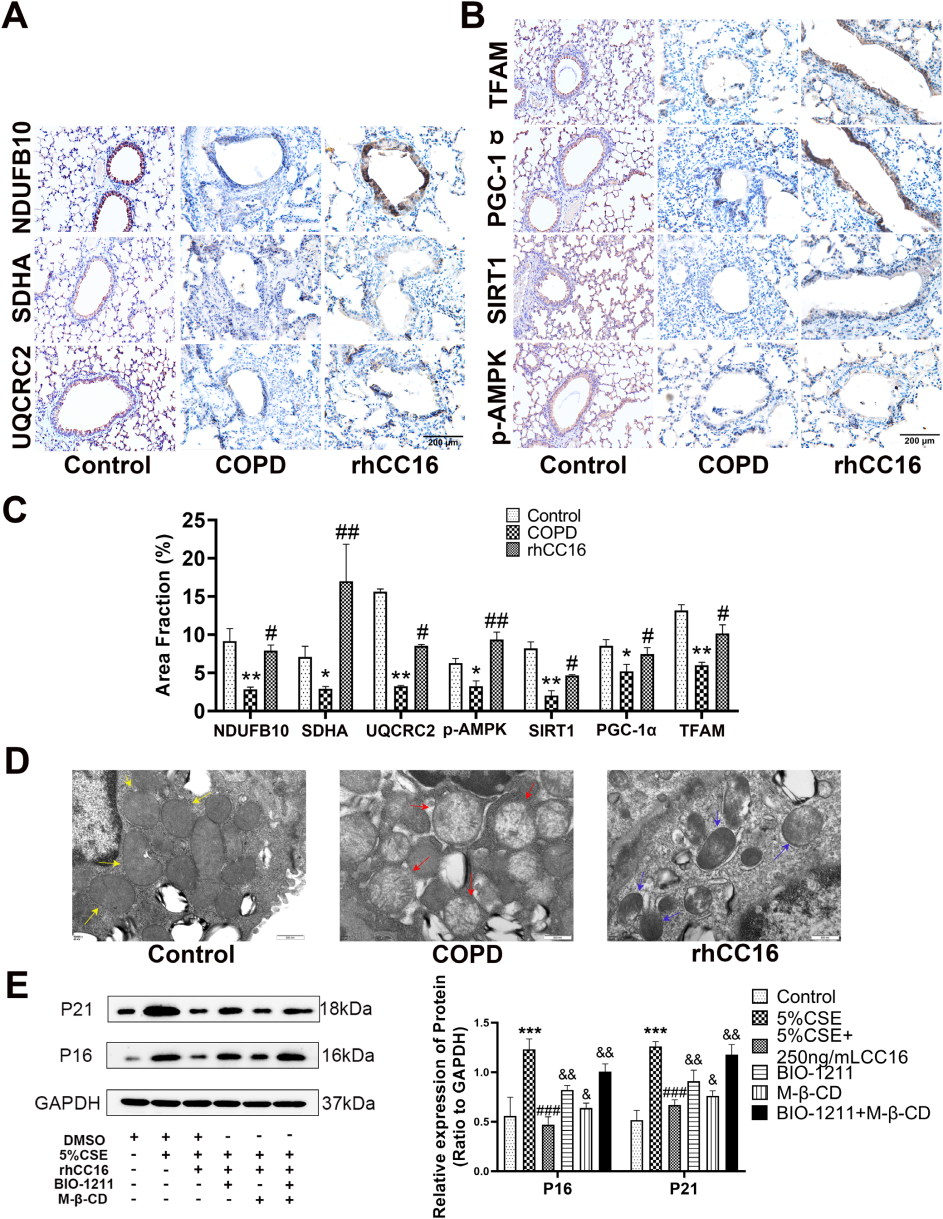
rhCC16 improved mitochondrial function via the AMPK/Sirt1/PGC‐1α/TFAM pathway both in vitro and in vivo. (A) Representative images of the oxidative phosphorylation complex I, complex II, and complex III proteins obtained via immunohistochemistry analysis of mouse lung tissue samples from the control, COPD, and rhCC16 treatment groups; scale bar: 200 μm. (B) Representative images of p‐AMPK, SIRT1, PGC‐1α, and TFAM obtained from immunohistochemical analysis of mouse lung tissue samples from the control, COPD, and rhCC16 treatment groups; scale bar: 200 μm. (C) Statistical bar chart plot for Figure A and Figure B. (D) Representative TEM images of mouse lung tissue samples from the control, COPD, and rhCC16 treatment groups; yellow arrows represent mitochondria with normal morphology; red arrows represent mitochondria with abnormal morphology and functional damage; blue arrows represent mitochondria with restored morphology and function. Scale bar: 500 nm. (E) The levels of p16 and p21 proteins in CSE‐stimulated or rhCC16‐treated BEAS‐2B cells treated with BIO‐1211 (10 μM) or M‐β‐CD (5 μM) were determined using Western blot. The data were presented as the mean ± SEM. *p < 0.05, **p < 0.01, and ***p < 0.001, compared to the control group; ^#^ *p* < 0.05, ^##^ *p* < 0.01, and ^###^ *p* < 0.001, compared to the 5% CSE group; ^&^ *p* < 0.05, ^&&^ *p* < 0.01, compared to the 5% CSE + rhCC16 group.

Next, lung tissue samples from the mice with COPD were fixed, paraffin embedded, and subjected to immunohistochemistry analysis. The results revealed that the levels of markers of mitochondrial oxidative phosphorylation (complex I: NDUFB10, complex II: SDHA, and complex III: UQCRC2) were decreased significantly in the lung tissue samples of these mice with COPD, while the levels of these three markers were significantly increased in the rhCC16‐treated mice (Figure 6A,C). Furthermore, the levels of p‐AMPK, Sirt1, PGC‐1‐α, and TFAM were elevated in the lung tissues of rhCC16‐treated COPD mice and decreased in those of mice with COPD (Figure 6B,C). Finally, the TEM results revealed that the mitochondria in the lungs of mice with COPD were swollen, with vacuolar‐like changes and reduced electron densities and that rhCC16 treatment restored mitochondrial structure to a state similar to that observed in control mice (Figure 6D). Collectively, these findings suggested that rhCC16 improved mitochondrial function via the AMPK/Sirt1/PGC‐1α–TFAM pathway both in vitro and in vivo.

### 3.7 rhCC16 Exerted Its Effect via Both Integrin α4β1‐Mediated and Clathrin‐Mediated Endocytosis

CC16 enters cells via clathrin‐mediated endocytosis [17] or by binding to the integrin α4β1 [27] to exert its effects. Therefore, BIO‐1211 (an inhibitor of α4β1 integrin) or methyl‐β‐cyclodextrin (M‐β‐CD, an inhibitor of clathrin) was used to identify the pathway involved in the effect of rhCC16 on BEAS‐2B cells. The results revealed that the inhibition of both clathrin and α4β1 integrin normalised the levels of P16 and P21, which were reduced upon rhCC16 treatment, although it appeared that α4β1 integrin played the main mediatory role in the present study (Figure 6E).

## 4 Discussion

Cigarette smoke (CS) contains complex components that are harmful to human health, as they lead to inflammatory reactions, oxidative stress, aging, apoptosis, and other damage to cells and lung tissues, which may result in the occurrence of COPD and promote its development [28, 29, 30]. In this context, the present study involved activating the PGC‐1α signalling pathway under the mediating effects of AMPK and Sirt1 to demonstrate that exogenous supplementation with rhCC16 effectively inhibited cigarette smoke extract (CSE)‐induced cellular senescence in vitro and CS‐induced lung COPD‐like pathological changes in mice.

CC16 is secreted mainly by non‐ciliated bronchial epithelial cells and is detectable in the respiratory tract, circulation, sputum, nose, and urine of individuals [9]. According to the literature, a decrease in the serum CC16 concentration is positively correlated with pulmonary function and negatively correlated with smoking and gold grade [31]. In addition, CC16 has been reported to be an independent risk factor for COPD and could also be used for predicting the progression of emphysema in patients with α‐1 antitrypsin deficiency during COPD [32]. Certain studies demonstrated that rhCC16 could limit the progression of emphysema in mice through a reduction in the activity of NF‐kappa B in CC16‐knockout lung tissues stimulated by CSE and the rescue of decreased Foxj1 expression [33]. The present study revealed that the addition of rhCC16 effectively improved lung function and lung COPD‐like changes in mice with COPD (Figure 2E,G; Table 2). These findings, along with the data reported in the literature, imply that rhCC16 addition is a potential therapeutic strategy against COPD.

Numerous studies have demonstrated that CSE can induce oxidative stress and the production and accumulation of ROS, thereby inducing COPD [34, 35, 36]. It is well recognised that ROS, as a byproduct of mitochondrial metabolism, are cleared through the antioxidant enzyme system [37]. Mitochondrial dysfunction, even without a reduction in mitochondrial capacity, can lead to the accumulation of ROS. Excessive ROS damage the structure and function of mitochondria and lead to a reduced capacity for oxidative phosphorylation dysfunction, which ultimately causes cell senescence and COPD [38, 39]. Mitochondrial dysfunction refers to disorders of mitochondrial biological function, abnormal accumulation of harmful subunits, and disorders of the mitochondrial‐dependent signalling pathway [40]. Stimulation of the respiratory system, either exogenously or endogenously, alters the metabolic pathway of mitochondrial oxidative phosphorylation in alveolar epithelial cells [41]. Changes in the mitochondrial metabolic pathway and mitochondrial dysfunction lead to greater mitochondria‐derived ROS (mtROS) levels and an increased degree of mitochondrial damage. In the present study, the accumulation of ROS produced upon CSE treatment led to cellular senescence and mitochondrial dysfunction. After exogenous supplementation with rhCC16, the accumulated ROS were eliminated. This process was achieved by salvaging the antioxidant enzyme system (Figure 4A–H). In addition, the present study revealed that rhCC16 rescued the damage caused by PGC‐1α and TFAM. Peroxisome proliferator‐activated receptor gamma cofactor‐1α (PGC‐1α) is the main transcriptional regulator of mitochondrial remodelling and biogenesis [42], and its regulation is closely influenced by several transduction effectors, such as adenosine monophosphate activated protein kinase (AMPK) and silent information regulator 1 (Sirt1), among others [43, 44].

AMPK may be activated by adverse conditions and stresses such as low glucose and hypoxia, after which the generated p‐AMPK actively regulates related signalling pathways, including the activation of autophagy pathways, the production of various antioxidant proteins, and the activation of PGC‐1α‐related signalling pathways [45, 46, 47]. Several recent studies have linked AMPK to mitochondrial function and cellular aging, and it is believed that the activation of AMPK plays a positive role in preventing mitochondrial damage and treating aging [48, 49, 50]. In the present study, rhCC16 significantly increased the expression of p‐AMPK and activated both PGC‐1α and TFAM. PGC‐1α promotes the expression of TFAM by binding to NRF1/2, and this activation of the PGC‐1α‐NRF1/2‐TFAM pathway facilitates mitochondrial DNA replication and protein synthesis, which ultimately leads to the production of new mitochondria [51]. PGC‐1α is also reported to regulate mitochondrial biogenesis through the regulation of NRF1/2, which subsequently regulates TFAM [52]. This intricate regulatory network ensures the proper functioning and renewal of mitochondria, which are essential for cellular energy production [51]. Sirt1 and AMPK are interrelated and mutually regulated and are also reported to share the common target molecule PGC‐1α [53, 54]. The findings of the present study confirmed that Dorsomorphin could inhibit the expression of p‐AMPK, as well as that of Sirt1, and the Sirt1 inhibitor Ex527 also inhibited the expression of Sirt1 and p‐AMPK. Both inhibitors inhibited the expression of PGC‐1α, which indicated that rhCC16 activates both AMPK and Sirt1 and then targets PGC‐1α to participate in the modulation of mitochondrial function. Moreover, the activation of AMPK increased the concentration of intracellular NAD+, thereby promoting the expression of Sirt1, which was consistent with previous reports [55]. The findings revealed an interaction between AMPK and Sirt1, which promoted the expression of PGC‐1α, thereby inhibiting cellular senescence [56, 57]. The animal experiments conducted in the present study also revealed that the activity of the AMPK/Sirt1‐PGC‐1α‐TFAM pathway was reduced in mice with COPD and that rhCC16 administration activated this pathway and mitigated COPD‐like symptoms.

The present study demonstrated that rhCC16 could inhibit cellular senescence and antagonise the development of COPD by activating the mitochondrial biogenesis pathway. The next step was to understand how exogenously supplemented rhCC16 acted on cells and exerted its biological effects. According to recent studies, rhCC16 functions by binding to integrin α4β1 [27]. As a small molecule protein, rhCC16 also functions via clathrin‐mediated endocytosis [17]. The present study revealed that rhCC16 exerted its effects mainly by binding to integrin α4β1, while only a part of its function was dependent on clathrin‐mediated endocytosis. In addition, after the two inhibitors were used simultaneously, it became more difficult for rhCC16 to exert its anti‐aging effect, which manifested as increased levels of the p16 and p21 proteins (Figure 6E). The β subunit tail of integrin contains a unique regulatory sequence that binds to the auxiliary proteins associated with clathrin‐mediated endocytosis to promote endocytosis [58]. Moreover, foreign substances may bind to β‐integrin and promote the recruitment of clathrin for internalisation [59]. This signifies that the integrin pathway and clathrin‐mediated endocytosis might be interconnected during the functioning of rhCC16. When α4β1 is inhibited by BIO‐1211, the binding of rhCC16 to α4β1 and the recruitment of clathrin by α4β1 are inhibited simultaneously, and the anti‐senescence effect of rhCC16 is minimal. On the other hand, when clathrin is inhibited by M‐β‐CD, endocytosis is impeded, while rhCC16‐α4β1 integrin binding remains unaffected. Another explanation could be that the affinity coefficient for the ligand–receptor interaction is high, and therefore, their binding requires less energy, making this binding convenient. Nevertheless, clathrin‐mediated endocytosis requires greater energy. Under CSE stimulation, the mitochondrial structure and function of BEAS‐2B cells are disrupted, hindering energy synthesis and generating inadequate energy for endocytosis. Accordingly, it was inferred that rhCC16 functions mainly by binding to integrins. Of course, further study will be performed using BIO‐1211 or M‐β‐CD on COPD mice to validate the same mechanism of rhCC16 in COPD in our next work.

In summary, rhCC16 exerts its anti‐senescence effects by activating the AMPK/Sirt1‐PGC‐1α‐TFAM pathway to promote mitochondrial biogenesis. In addition, the function of rhCC16 is dependent on both α4β1 integrin binding and clathrin‐mediated endocytosis.

This article also has certain limitations, as the arguments regarding ROS only appear in vitro experiments and are not concurrently discussed in vivo experiments. Additionally, measuring the concentration of intracellular fluorescent probes is crucial for interpreting results. When the rate of oxidant production inside the cell remains constant, an increase or decrease in probe uptake will alter the quantity of the products formed [60]. Therefore, ROS measurements using high‐resolution respirometry or spin traps are preferable. Furthermore, as our research progressed, we found that rhCC16 may simultaneously affect autophagy through the AMPK pathway, thereby jointly influencing mitochondrial function. However, this manuscript does not investigate issues related to autophagy further, which may be the direction of our next research step.

## Author Contributions

**Ying‐jie Ren:** data curation (lead), investigation (equal), methodology (equal), resources (equal), writing – original draft (lead). **Tian‐qi Sun:** conceptualization (supporting), data curation (supporting), investigation (equal), methodology (equal), writing – original draft (supporting). **Yu Lu:** data curation (equal), investigation (equal), methodology (equal). **Dan‐Li Liu:** conceptualization (supporting), data curation (supporting), investigation (equal), methodology (equal), resources (equal), writing – review and editing (equal). **Rui Gao:** data curation (supporting), investigation (supporting), resources (supporting), writing – original draft (supporting). **Ting Li:** data curation (supporting), formal analysis (supporting), project administration (supporting), writing – review and editing (supporting). **Min Guo:** resources (equal), supervision (supporting), validation (equal), visualization (equal). **Qing‐hua Liu:** supervision (supporting), validation (supporting), visualization (supporting). **Hai‐long Wang:** conceptualization (lead), methodology (equal), project administration (equal), supervision (equal), writing – review and editing (equal). **Min Pang:** conceptualization (equal), formal analysis (equal), funding acquisition (lead), methodology (supporting), resources (equal), supervision (equal).

## Conflicts of Interest

The authors declare no conflicts of interest.

## Supporting information


**Figure S1.** [Western blot] The concentration of CSE (A, B) or rhCC16 (C, D), determined using the CCK‐8 assay. The level of the P16 protein was determined through western blot to determine the appropriate dose of rhCC16 for establishing the cellular senescence model. The data are presented as the mean ± SEM. **p < 0.01 and ***p < 0.001, compared to 0% or 0 ng/mL.

**Figure S2.** (A) Western blot detection of the apoptosis markers caspase3 and PARP in the presence of different concentrations of CSE. (B) Representative images of Hoechst33258 staining after treatment with different concentrations of CSE.

**Figure S3.** (A) The other SASP‐related mRNAs were assayed using RT–qPCR. (B) Statistical analysis of the Western blot data presented in Figure 5E [Western blot]. (C) Statistical analysis of the western blot data were presented in Figure 5F. The data are presented as the mean ± SEM. *p < 0.05, **p < 0.01 and ***p < 0.001 compared to the control group; ^#^ *p* < 0.05, ^##^ *p* < 0.01 and ^###^ *p* < 0.001 compared to the 5% CSE group; ^&^ *p* < 0.05 and ^&&^ *p* < 0.01, compared to the 5% CSE + rhCC16 group.

## Data Availability

The data that support the findings of this study are available from the corresponding author upon reasonable request.
