# Supplementary figures and images for: rhCC16 Suppresses Cellular Senescence and Ameliorates COPD‐Like Symptoms by Activating the AMPK/Sirt1‐PGC‐1‐α‐TFAM Pathway to Promote Mitochondrial Function
Source: J Cell Mol Med. 2025 Apr 21;29(8):e70566. doi: 10.1111/jcmm.70566 (PMC12011551; doi:10.1111/jcmm.70566)

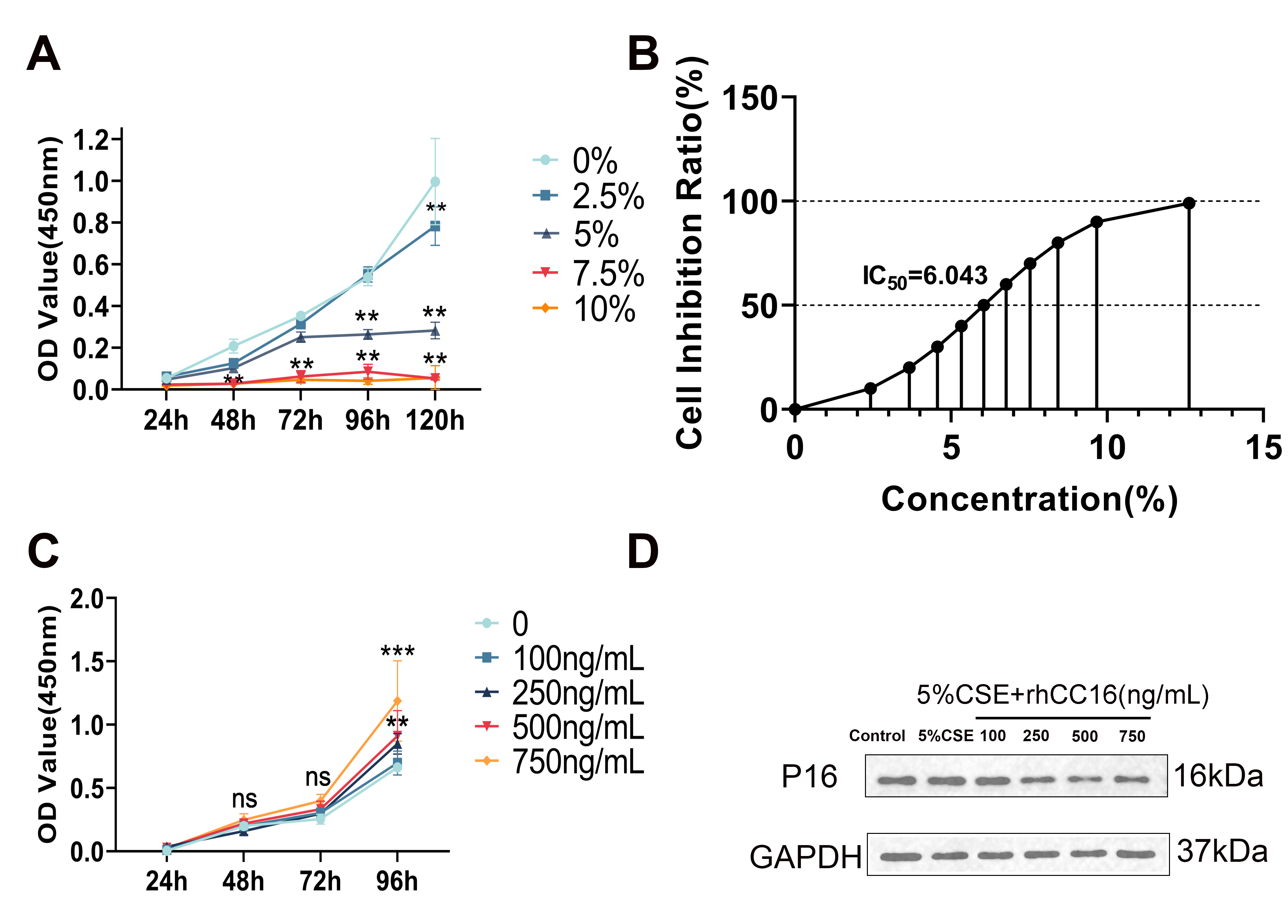

Supplement: Supplementary file 1 — Figure S1. [Western blot] The concentration of CSE (A, B) or rhCC16 (C, D), determined using the CCK‐8 assay. The level of the P16 protein was determined through western blot to determine the appropriate dose of rhCC16 for establishing the cellular senescence model. The data are presented as the mean ± SEM. **p < 0.01 and ***p < 0.001, compared to 0% or 0 ng/mL. [file JCMM-29-e70566-s002.tif]

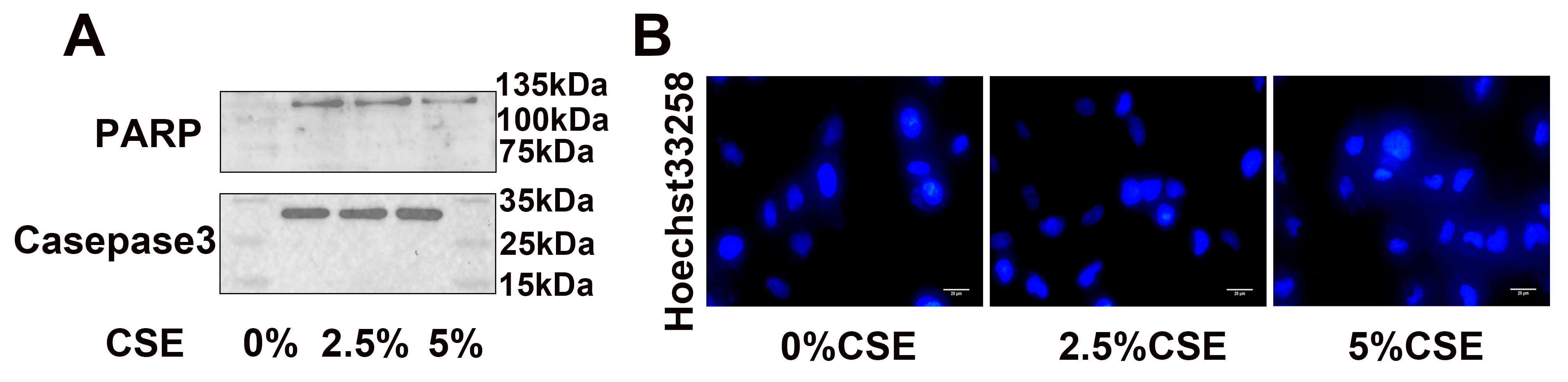

Supplement: Supplementary file 2 — Figure S2. (A) Western blot detection of the apoptosis markers caspase3 and PARP in the presence of different concentrations of CSE. (B) Representative images of Hoechst33258 staining after treatment with different concentrations of CSE. [file JCMM-29-e70566-s001.tif]

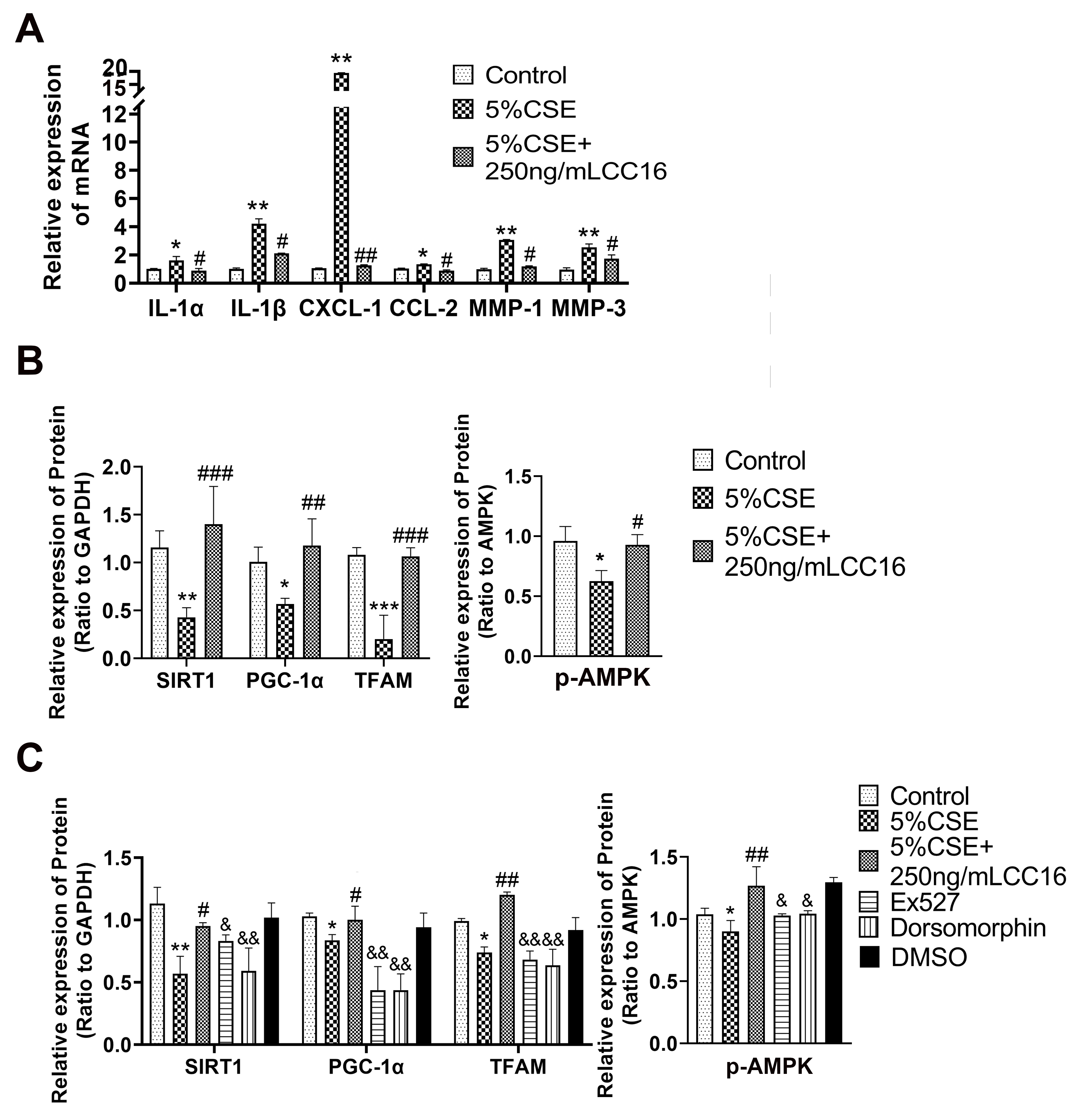

Supplement: Supplementary file 3 — Figure S3. (A) The other SASP‐related mRNAs were assayed using RT–qPCR. (B) Statistical analysis of the Western blot data presented in Figure 5E [Western blot]. (C) Statistical analysis of the western blot data were presented in Figure 5F. The data are presented as the mean ± SEM. *p < 0.05, **p < 0.01 and ***p < 0.001 compared to the control group; # p < 0.05, ## p < 0.01 and ### p < 0.001 compared to the 5% CSE group; & p < 0.05 and && p < 0.01, compared to the 5% CSE + rhCC16 group. [file JCMM-29-e70566-s003.tif]
